# Molecular Detection of Potato Viruses in Bangladesh and Their Phylogenetic Analysis

**DOI:** 10.3390/plants9111413

**Published:** 2020-10-22

**Authors:** Mamun-Or Rashid, Ying Wang, Cheng-Gui Han

**Affiliations:** State Key Laboratory for Agrobiotechnology and Key Laboratory of Pest Monitoring and Green Management, MOA, China Agricultural University, Beijing 100193, China; mamun_1961@yahoo.com (M.-O.R.); yingwang@cau.edu.cn (Y.W.)

**Keywords:** potato viruses, Bangladesh, RT-PCR, virus identification, phylogenetic analysis

## Abstract

Potato (*Solanum tuberosum*) is a major food source in the whole world including Bangladesh. Viral diseases are the key constraint for sustainable potato production by reducing both quality and quantity. To determine the present status of eight important potato viruses in Bangladesh, tuber samples were collected from three major potato growing regions (Munshiganj, Jessore and Bogra districts) in January–February 2017 and February 2018. Reverse transcription polymerase chain reaction (RT-PCR) with coat protein (CP)-specific primers were used to amplify CP sequences of the respective viruses, and confirmed by sequencing, which were deposited in the GenBank. Results indicated that the tuber samples were subjected to *Potato leafroll virus* (PLRV), *Potato virus X* (PVX), *Potato virus Y* (PVY), *Potato virus S* (PVS), *Potato virus H* (PVH), *Potato aucuba mosaic virus* (PAMV) and *Potato virus M* (PVM) infection, whereas mixed infections were very common. Phylogenetic analysis revealed that the PLRV from this study was closely related to a Canadian and a Chinese isolate, respectively; PVX was closely related to a Canadian and a Chinese isolate, respectively; PVY was closely related to a Chinese isolate; PVS was closely related to a Chinese and an Iranian isolate, respectively; PAMV was closely related to a Canadian isolate; PVH was closely related to a Huhhot isolate of China; and PVM was closely related to an Indian and an Iranian isolate, respectively. As far as we know, PAMV in this study is the first report in Bangladesh. These findings will provide a great scope for appropriate virus control strategies to virus free potato production in Bangladesh.

## 1. Introduction

Potato, the power house of energy, is the principal vegetable crop in Bangladesh [1]. In 2018, Bangladesh produces ~9,744,412 tonnes of potato from ~477,419 ha of land, holding the sixth position in the world [2]. In spite of using high-yielding varieties and improved cultivation methods, till now potato production per unit area is not up to the mark due to several biotic and abiotic factors including viruses [3]. As viruses are ubiquitous in nature, they are assumed as a major barrier for potato cultivation, particularly during mixed infection [4,5,6]. Worldwide, over 50 virus species have been recognized that affect potato production by reducing tuber yield of above 50% in the case of single infection, and which can reach beyond 80% during mixed infections, causing losses of over 50 billion euros annually [6,7,8]. In Bangladesh, a total of seven potato viruses, *Potato leafroll virus* (PLRV; family: Leutoviridae, genus: Polerovirus), *Potato virus X* (PVX; family: Alphaflexiviridae, genus: Potexvirus), *Potato virus Y* (PVY; family: Potyviridae, genus: Potyvirus), *Potato virus S* (PVS; family: Betaflexiviridae, genus: Carlavirus), *Potato virus H* (PVH; family: Betaflexiviridae, genus: Carlavirus), *Potato virus M* (PVM; family: Betaflexiviridae, genus: Carlavirus) and *Potato virus A* (PVA; family: Potyviridae, genus: Potyvirus) have been reported so far [9,10,11,12]. Among them, PLRV, PVX and PVY are predominant, causing an annual-yield losses of 15–78% [13,14,15]. Besides, *Potato aucuba mosaic* virus (PAMV; family: Alphaflexiviridae, genus: Potexvirus) is an emerging potato virus worldwide, especially in Southeast Asia [6,16,17]. Availability of virus-free seed tuber is a major obstruction for sustainable potato farming in Bangladesh [7]. However, documentation of actual disease-causing agent is crucial for proper management program [18]. Detection of potato viruses at the initial stage is one of the best ways to control the disease development and virus free seed potato production [8]. The authentic detection of viruses in seed lot followed by destruction of infected tubers is an effective control strategy that inhibits virus infection in field [19]. Accuracy, rapidity and specificity are the key factors for virus documentation. Regular methods of virus documentation comprising susceptible indicator hosts inoculation, electron microscopy, ELISA and visual observation are not reliable for virus documentation in dormant potato [20]. Reverse transcription polymerase chain reaction (RT-PCR) detection is an appropriate and more commonly used alternative method to overcome these difficulties [21,22]. In addition, RT-PCR technique is 10^2^ to 10^5^ times more sensitive in comparison to traditional ELISA technique for potato virus detection [23]. Although, the success of PCR detection mainly relies upon the specificity of designed primer pairs. Reverse transcription loop-mediated isothermal amplification (LAMP), a highly sensitive technique of virus detection has recently been developed to detect PLRV, PVX and PVY [24,25,26]. Next-generation sequencing (NGS) technologies has become more popular for the documentation and characterization of novel viruses and viroids in the last few years [27,28,29]. However, PCR-based technologies are often used in most diagnostic laboratories for virus detection due to its accuracy, specificity, sensitivity and relatively low cost [30].

>In the present study, RT-PCR based detection followed by subsequent sequencing was performed to identify and characterize eight important potato viruses (PLRV, PVX, PVY, PVS, PVH, PAMV, PVM and PVA) in three key potato growing regions (Munshiganj, Jessore and Bogra districts) of Bangladesh and also accomplish their relative incidence. Additionally, phylogenetic association with formerly reported virus isolates from the National Center for Biotechnology Information (NCBI) database was also analyzed. Findings from this study will provide an excellent scope for future research on the biological features of these viruses, including the effects on yield and quality, host range and distribution, and also for virus free potato production.

## 2. Results

### 2.1. Detection of Potato Viruses

Rapid and exact detection of viruses are pre-requisite for virus-free seed potato production, and planting of these virus-free potato seed is an important approach to control potato viruses in field to enhance the commercial potato production [31]. RT-PCR based detection followed by subsequent sequencing were performed in this study to determine the present status of eight important potato viruses in three major potato growing regions (Munshiganj, Jessore and Bogra districts) of Bangladesh. The RT-PCR results showed that the PLRV, PVX, PVY, PVS, PVH, PAMV and PVM were associated with the collected tuber samples (Figure S1). Whereas, PVM was only observed in Bogra, PVH and PAMV were observed both in Jessore and Bogra, and PLRV, PVX, PVY and PVS were observed in all the three regions (Table 1). In addition, detection results revealed that mixed infections were frequent in collected tuber samples (Table 1). Interestingly, we did not observe any tuber sample was infected by only one virus. They either free from viruses or infected by two or multiple viruses. However, further investigation is needed on this issue in the future. More interestingly, PVA was absent in the tested tuber samples in the present study.

Sequence analysis from this study with the BLASTn server of NCBI confirmed the presence of PLRV, PVX, PVY, PVS, PVH, PAMV and PVM (Table 2). All these sequences were deposited to NCBI GeneBank. As far as we know, this is the first report of PAMV in Bangladesh.

Comparison of viral sequences found in Bangladesh showed that PLRV isolates shared a maximum 95.69% nucleotide (nt) identity with each other; PVX shared a maximum 96.36%, 97.06% and 99.30% nt identity with each other; PVS shared a maximum 93.79% nt identity with each other; PVM shared a maximum 80.59% nt identity with each other; and PAMV shared a maximum 96.93%, 97.47% and 97.60% nt identity with each other. Comparison of viral sequences from the present study with the respective viral sequences from the GenBank database further exhibited that PLRV from this study shared a maximum 95.52% and 100% nt identity with a Chinese isolate (GenBank accession no. MF062487) and a Canadian isolate (GenBank accession no. D13954), respectively; PVX shared a maximum 98.31%, 99.72% and 98.59% nt identity with a Scottish isolate (GenBank accession no. GU144353), a Shandong isolate (GenBank accession no. AF528555) and a Heilongjiang isolate of China (GenBank accession no. GU373815), respectively; PVY shared a maximum 99.38% nt identity with a Chinese isolate (GenBank accession no. KC296825); PVS shared a maximum 98.87% and 95.14% nt identity with a Chinese isolate (GenBank accession no. AY512653) and an Iranian isolate (GenBank accession no. MH159208), respectively; PVH shared a maximum 100% nt identity with a Huhhot isolate of China (GenBank accession no. HM584819); PAMV shared a maximum 97.07–97.60% nt identity with a Canadian isolate (GenBank accession no. S73580); and PVM shared a maximum 93.68% and 82.24% nt identity with an Indian isolate (GenBank accession no. KJ473993) and an Iranian isolate (GenBank accession no. KC129092), respectively. As PVH-CP sequence exhibited 100% nt similarity with a Huhhot isolate of China, we executed an additional RT-PCR with a primer pair encoding the triplegene block-1 region, whereas the sequence (GenBank accession no. MH932394) shared a maximum 99.86% nt identity with the same isolate.

### 2.2. Phylogenetic Analysis

**Figure 1:**
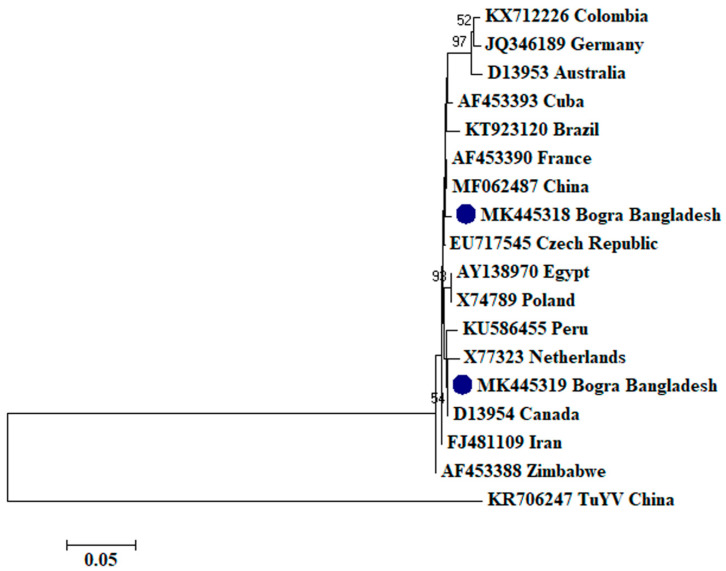
Phylogenetic analysis of *Potato leafroll virus* detected from Bangladesh based on the coat protein gene sequences (nt) of different selected isolates from the NCBI database. The phylogenetic tree was generated by MEGA7 using neighbor-joining method with 1000 bootstrap replicates, and only values >50% are shown at the nodes. An isolate of *Turnip yellows virus* (TuYV) from China was used as an outgroup.

**Figure 2:**
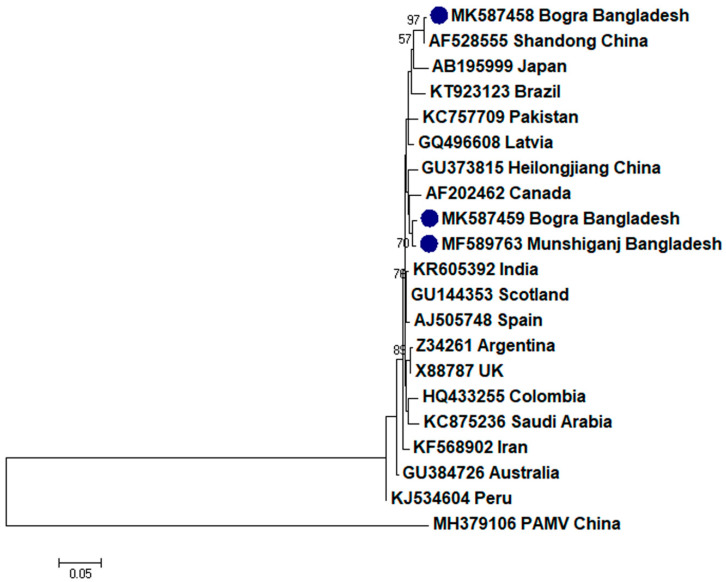
Phylogenetic analysis of *Potato virus X* detected from Bangladesh based on the coat protein gene sequences (nt) of different selected isolates from the NCBI database. The phylogenetic tree was generated by MEGA7 using neighbor-joining method with 1000 bootstrap replicates, and only values >50% are shown at the nodes. An isolate of *Potato aucuba mosaic virus* (PAMV) from China was used as an outgroup.

**Figure 3:**
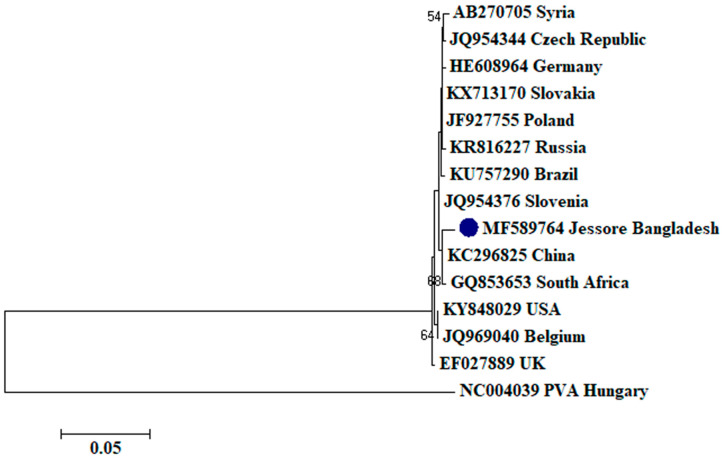
Phylogenetic analysis of *Potato virus Y* detected from Bangladesh based on the coat protein gene sequences (nt) of different selected isolates from the NCBI database. The phylogenetic tree was generated by MEGA7 using neighbor-joining method with 1000 bootstrap replicates, and only values >50% are shown at the nodes. An isolate of *Potato virus A* (PVA) from Hungary was used as an outgroup.

**Figure 4:**
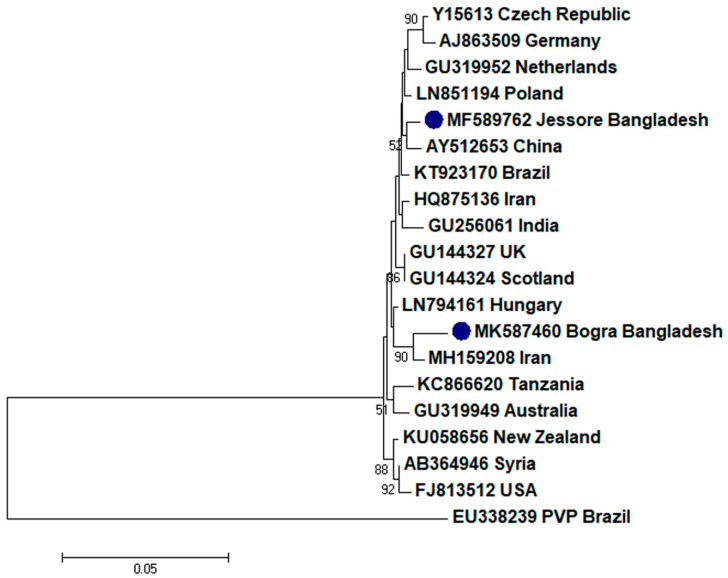
Phylogenetic analysis of *Potato virus S* detected from Bangladesh based on the coat protein gene sequences (nt) of different selected isolates from the NCBI database. The phylogenetic tree was generated by MEGA7 using neighbor-joining method with 1000 bootstrap replicates, and only values >50% are shown at the nodes. An isolate of *Potato virus P* (PVP) from Brazil was used as an outgroup.

**Figure 5:**
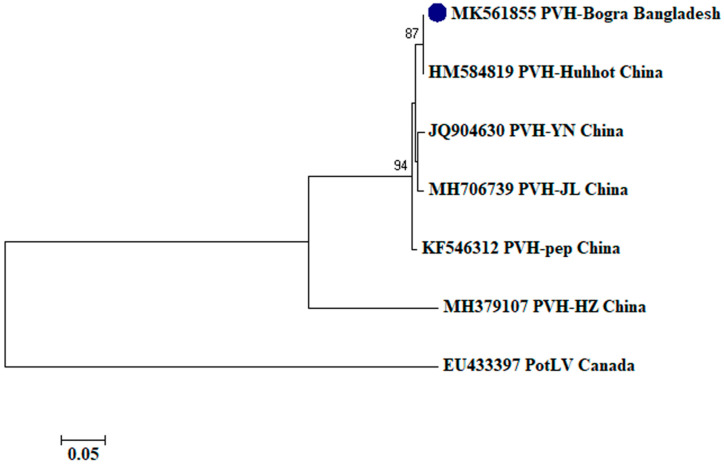
Phylogenetic analysis of *Potato virus H* detected from Bangladesh based on the coat protein gene sequences (nt) of different selected isolates from the NCBI database. The phylogenetic tree was generated by MEGA7 using neighbor-joining method with 1000 bootstrap replicates, and only values >50% are shown at the nodes. An isolate of *Potato Latent virus* (PotLV) from Canada was used as an outgroup.

**Figure 6:**
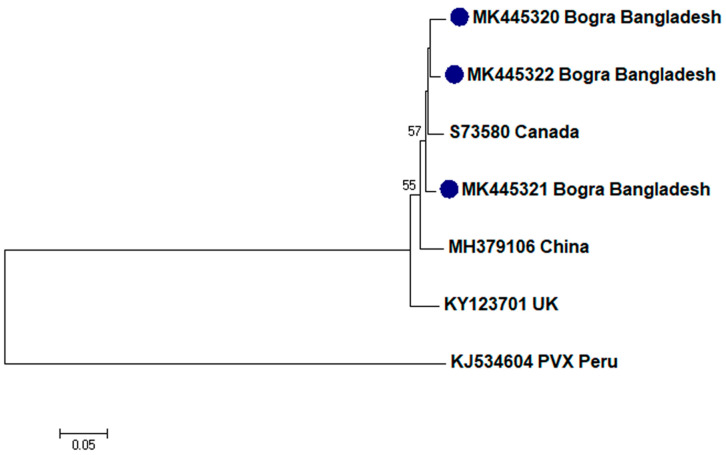
Phylogenetic analysis of *Potato aucuba mosaic virus* detected from Bangladesh based on the coat protein gene sequences (nt) of different selected isolates from the NCBI database. The phylogenetic tree was generated by MEGA7 using neighbor-joining method with 1000 bootstrap replicates, and only values >50% are shown at the nodes. An isolate of *Potato virus X* (PVA) from Peru was used as an outgroup.

**Figure 7:**
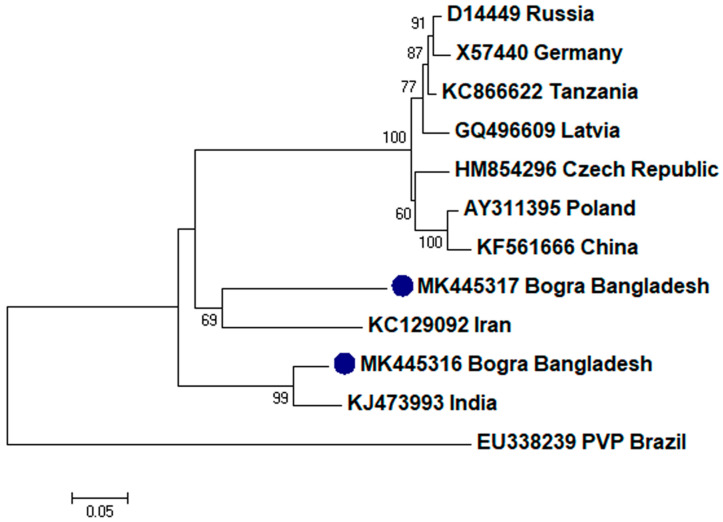
Phylogenetic analysis of *Potato virus M* detected from Bangladesh based on the coat protein gene sequences (nt) of different selected isolates from the NCBI database. The phylogenetic tree was generated by MEGA7 using neighbor-joining method with 1000 bootstrap replicates, and only values >50% are shown at the nodes. An isolate of *Potato virus P* (PVP) from Brazil was used as an outgroup.

To assess the evolutionary relationship, nucleotide sequences from this study and existing sequences in NCBI database of respective viruses were aligned by ClustalW [32]. Here, ingroups were selected from the same species from different countries based on the closely related CP sequences to the respective viruses and outgroups were selected from the same family to the respective ingroup virus species containing enough homologous sites to the respective ingroup virus species. Neighbor-joining phylogenetic trees [33] using these aligned sequences showed that PLRV from this study was closely related to a Chinese isolate (GenBank accession no. MF062487) and a Canadian isolate (GenBank accession no. D13954), respectively (Figure 1); PVX was closely related to a Canadian isolate (GenBank accession no. AF202462) and a Shandong isolate of China (GenBank accession no. AF528555), respectively (Figure 2); PVY was closely related to a Chinese isolate (GenBank accession no. KC296825; Figure 3); PVS was closely related to a Chinese isolate (GenBank accession no. AY512653) and an Iranian isolate (GenBank accession no. MH159208), respectively (Figure 4); PVH was closely related to a Huhhot isolate of China (GenBank accession no. HM584819; Figure 5); PAMV was closely related to a Canadian isolate (GenBank accession no. S73580; Figure 6); and PVM was closely related to an Indian isolate (GenBank accession no. KJ473993) and an Iranian isolate (GenBank accession no. KC129092), respectively (Figure 7).

## 3. Discussion

Viruses are the principal threat for potato production all over the world, including Bangladesh [5,10,34]. As the availability of virus-free seed tuber is the main barrier for increased potato production in Bangladesh, documentation of exact viruses in the seed lot as well as in the field is needed to enhance the potato production. However, a few number of studies has been performed on molecular detection and infection status of potato viruses in Bangladesh. Therefore, in this study, RT-PCR based detection followed by subsequent sequencing were performed to determine the present status of eight important potato viruses in three major potato growing regions (Munshiganj, Jessore and Bogra districts) of Bangladesh. Detection results showed that the tuber samples were associated with PLRV, PVX, PVY, PVS, PVH, PAMV and PVM, whereas PVA was absent. Among them, PLRV, PVX, PVY, PVS, PVH, PVM and PVA have been reported so far in Bangladesh [9,10,11,12], and as far as we know, this is the first report of PAMV in Bangladesh. In addition, detection results further demonstrated that mixed infection was very common, consistent with the earlier findings [4,35,36,37]. Although, single infection by PVX is not so damaging, however co-infection with other viruses (PVY, PVA and PVS) may produce vast yield losses [38]. Mixed infection of PLRV and PVY can generate huge economic losses by reducing tuber size and quality rather than solitary infection of PLRV or PVY [37]. PLRV, PVX and PVY are more destructive potato viruses in Bangladesh, responsible for an annual-yield losses of 15–78% [13,14,15]. Generally, single infection by PVA or PVH or PVM or PVS are symptomless and difficult to characterize, whereas various symptoms including chlorotic spot, rugosity and mottling of leaves, stunting and premature leaf dropping may observe during mixed infection of these viruses depending on the susceptibility of potato cultivars and virus isolates [39,40,41]. Zhang et al. [36] reported that co-infection of PLRV, PVX and PVY produced 1.25% infection, and PLRV, PVX, PVY and PVA produced 0.31% infection, respectively. Stevenson [35] also showed that mixed infection of PVX with PVA or PVY increased yield loss up to 45%, PVS with PVX or PVM increased yield loss up to 40%. However, PAMV is an emerging potato virus worldwide, especially in Southeast Asia [6,16,17], and in our study, we also detect PAMV in the collected tubers. As far as we know, this is the first report of PAMV in Bangladesh.

Phylogenetic analysis denotes various important information including the degree of relationship among genera, species and strains, diversity of geographical isolates, and the origin and evolution of plant viruses [42,43]. An appropriate selection of ingroups and outgroups are crucial steps in phylogenetic analysis as they significantly impact on tree topology and root position [44,45]. Ingroups should be a group of closely related species, whereas, outgroups should be distantly related to the ingroups but should have enough homologous sites to confirm that the tree is constructed upon comparison of homologous sites [46]. In this research, ingroups were selected from the same species from different countries based on the closely related CP sequences to the respective viruses and outgroups were selected from the same family to the respective ingroup virus species containing enough homologous sites to the respective ingroup virus species to assess the evolutionary relationship. Phylogenetic analysis confirmed that PLRV from this study was closely related to a Canadian and a Chinese isolate, respectively; PVX was closely related to a Canadian and a Chinese isolate, respectively; PVY was closely related to a Chinese isolate; PVS was closely related to a Chinese and an Iranian isolate, respectively; PAMV was closely related to a Canadian isolate; PVH was closely related to a Huhhot isolate of China; and PVM was closely related to an Indian and an Iranian isolate, respectively. This findings will be a foundation for further molecular evolutionary study on potato viruses in Bangladesh.

Globally, a number of researchers used identical potato virus identification techniques to our study [9,10,38,40,44,47,48,49]. However, as NGS are efficient methods for characterization of novel viruses [27,28,29], a number of novel viruses can be identified by using NGS from the collected tuber samples in the future.

## 4. Materials and Methods

### 4.1. Collection of Potato Tuber Samples

Potato tuber samples (Cultiver: Diamant and Lalpakri) from the three major potato growing regions of Bangladesh, Munshiganj (23°50′ N; 90°41′ E), Jessore (23°17′ N; 89°21′ E) and Bogra (24°85′ N; 89°37′ E) districts were collected at harvesting stage in January–February 2017 and February 2018. Altogether, a total of 220 tuber samples (50 tubers from Munshiganj, 50 tubers from Jessore and 120 tubers from Bogra districts) were picked randomly from 44 small fields (10 from Munshiganj, 10 from Jessore and 24 from Bogra districts) taking five tubers from each field for further RT-PCR detection.

### 4.2. RT-PCR Detection

The occurrence of eight economically important potato viruses in three major potato growing regions (Munshiganj, Jessore and Bogra districts) of Bangladesh were determined by RT-PCR and confirmed by successive sequencing. Total RNAs from all the five tubers of 44 sample lots were extracted using the method described formerly by Han et al. [50]. Briefly, 0.2 g tuber scrap from each sample was crumbled in liquid nitrogen followed by adding 600 µL of phenol–chloroform admixture and 630 µL of extraction buffer (20 mM Tris–HCl with pH 7.8, 1% sodium dodecyl sulfate, 200 mM sodium chloride, and 5 mM EDTA) with uninterrupted homogenization. Supernatants were collected after centrifugation. Equal volume of 4 M lithium chloride was used to precipitate the RNAs. Pelleted RNAs were collected after washing with chilled 75% ethanol and chilled 100% ethanol, and dissolved in diethyl pyrocarbonate-treated water to make the final volume of 40 µL.

The cDNAs were synthesized using Moloney murine leukemia virus (M-MLV) reverse transcriptase (Promega, Madison, WI, USA) as described by Khine et al. [51]. These cDNAs were used for further PCR amplification, accomplished as described earlier by Zhang et al. [52] with specific primer pairs designed based on the conserved CP-encoding sequence of the respective viruses (Table 3). Amplified PCR compounds were electrophoresed through 1.0% agarose gel encompassing ethidium bromide and envisaged by UV illumination (Gel Doc XR + Imaging System; Bio-Rad, Hercules, CA, USA).

### 4.3. Cloning and Sequence Analysis

AxyPrepTM DNA gel extraction kit (Axygen, CA, USA) was used to purify amplified products from the agarose gel. The purified PCR harvests were ligated to pMD19-T (Simple) vector (TaKaRa, Shiga, Japan) following the manufactures instruction and subsequently transformed into the competent cell of *Escherichia coli* strain MC1022 (a gift from Dr. Salah Bouzoubaa, University of Strasbourg, Strasbourg, France) as described earlier by Sambrook et al. [53]. Recombinant plasmids were confirmed by PCR and validated by successive sequencing of three positive clones of each plasmid (Genscript Biological Science, Nanjing, China) from each region of Bangladesh. Gene sequences from this study were scrutinized by DNAMAN version 6.0 (LynnonBiosoft, QC, Canada). Homology of the expected gene sequences was determined by the BLASTn server of NCBI.

### 4.4. Phylogenetic Analysis

Sequences from this study were aligned with related available sequences from the NCBI database by ClustalW [32]. Phylogenetic studies were accomplished by MEGA version 7 [33].

## 5. Conclusions

Viruses are major constraint for global potato production, including Bangladesh. Along with the expanding global trade, chance to migrate potato viruses is mounting day-by-day. Consequently, rapid and reliable potato virus detection techniques are mandatory to stop these migration as well as for virus free seed potato production. This study confirmed the presence of PLRV, PVX, PVY, PVS, PVH, PAMV and PVM in the three major potato growing regions (Munshiganj, Jessore and Bogra districts) of Bangladesh. In addition, virus incidence and their evolutionary relationship were also demonstrated here. As far as we know, this is the first report of PAMV in Bangladesh. Our findings will provide a base for further research on the biological features of the above-mentioned viruses, including the effects on yield and quality, host range, distribution, and also to develop appropriate management strategies for virus free potato production.

## Figures and Tables

**Table 1:** Incidence of potato viruses in 220 tuber samples collected from three major potato growing regions (Munshiganj, Jessore and Bogra districts) of Bangladesh in 2017–2018.

Sample Collected Regions	No. of Collected Tuber Samples	Associated Virus Name	RT-PCR Detection	
			No. of Infected Tubers	Infection Ratio (%)
Munshiganj	50	PLRV/PVX	7	14
		PVX/PVS	5	10
		PLRV/PVX/PVS	7	14
		PLRV/PVX/PVY	6	12
		PLRV/PVY/PVS	6	12
Jessore	50	PLRV/PVX	3	6
		PVX/PVS	5	10
		PVX/PAMV	6	12
		PLRV/PVX/PVS	3	6
		PLRV/PVY/PVS	5	10
		PVX/PVH/PAMV	4	8
		PLRV/PVX/PVH/PAMV	5	10
		PVX/PVS/PVH/PAMV	3	6
		PLRV/PVX/PVS/PVH/PAMV	1	2
Bogra	120	PVX/PAMV	4	3.33
		PLRV/PVX/PVS	5	4.17
		PLRV/PVX/PVY	4	3.33
		PLRV/PVY/PVS	4	3.33
		PVX/PVH/PAMV	11	9.17
		PLRV/PVX/PVH/PAMV	15	12.50
		PVX/PVS/PVH/PAMV	7	5.83
		PLRV/PVX/PVS/PVH/PAMV	4	3.33
		PLRV/PVX/PVS/PVH/PAMV/PVM	10	8.33

**Table 2:** Virus sequences obtained from three major potato growing regions (Munshiganj, Jessore and Bogra districts) of Bangladesh in this study.

Virus Name	GenBank Accession No.	Sample Collected Regions		
		Bogra	Munshiganj	Jessore
PLRV	MK445318	+	-	-
PLRV	MK445319	+	+	+
PVX	MK587458	+	-	-
PVX	MK587459	+	-	-
PVX	MF589763	-	+	+
PVY	MF589764	+	+	+
PVS	MF589762	-	+	+
PVS	MK587460	+	-	-
PVH	MK561855	+	-	+
PAMV	MK445320	+	-	+
PAMV	MK445321	+	-	-
PAMV	MK445322	+	-	-
PVM	MK445316	+	-	-
PVM	MK445317	+	-	-

+: Present; -: Absent.

**Table 3:** Primers used for RT-PCR detection in this study.

Primer	Sequence (5′ to 3′)
PLRV-CPF	ATGAGTACGGTCGTGGTTAA
PLRV-CPR	CTATTTGGGGTTTTGCAAAG
PVX-CPF	TAGCCACAACACAGGCCACAG
PVX-CPR	TTATGGTGGTGGGAGAGTGACAA
PVY-CPF	ACGTCCAAAATGAGAATGCC
PVY-CPR	TGGTGTTCGTGATGTGACCT
PVS-CPF	ATGCCGCCCAAACCGGAT
PVS-CPR	TCATTGGTTGATCGCATTAC
PVH-CPF	ATGGATGTTGCTACTTC
PVH-CPR	ATTGCCGCTTGTTATTC
PAMV-CPF	ATGGTTGATTCTAAGAAAAC
PAMV-CPR	TTAGGATGGTGGTGCCATGA
PVM-CPF	ATGGGAGATTCAACTAAGAA
PVM-CPR	CTTCATTTGTTATTCGACTT
PVA-CPF	AGCCGAAACTCTTGATGCAA
PVA-CPR	GCACTTCGGTTACACCCCCT
PVH-TGB1F	AACTGCAGATGGATGTGCTGGTAG
PVH-TGB1R	CGGGATCAGGCGGCGGTGAAAGTG

## Supplementary Materials

The following are available online at https://www.mdpi.com/2223-7747/9/11/1413/s1, Figure S1: Detection of potato viruses in tuber samples collected from Bangladesh by RT-PCR. Lane -: extract from a healthy potato tuber (negative control), Lanes 1–12: extracts from the individual tuber sample collected from different locations. (A) *Potato leafroll virus*; (B) *Potato virus X*; (C) *Potato virus Y*; (D) *Potato virus S*; (E) *Potato virus H*; (F) *Potato aucuba mosaic virus*; (G) *Potato virus M* and (H) *Potato virus A*.
