# Supplementary material for: Molecular Detection of Potato Viruses in Bangladesh and Their Phylogenetic Analysis
Source: Plants (Basel). 2020 Oct 22;9(11):1413. doi: 10.3390/plants9111413 (PMC7690588; doi:10.3390/plants9111413)
Supplement: Supplementary file 1 [file plants-09-01413-s001.pdf]

plants

MDPI

Article

Molecular Detection of Potato Viruses in Bangladesh and Their Phylogenetic Analysis

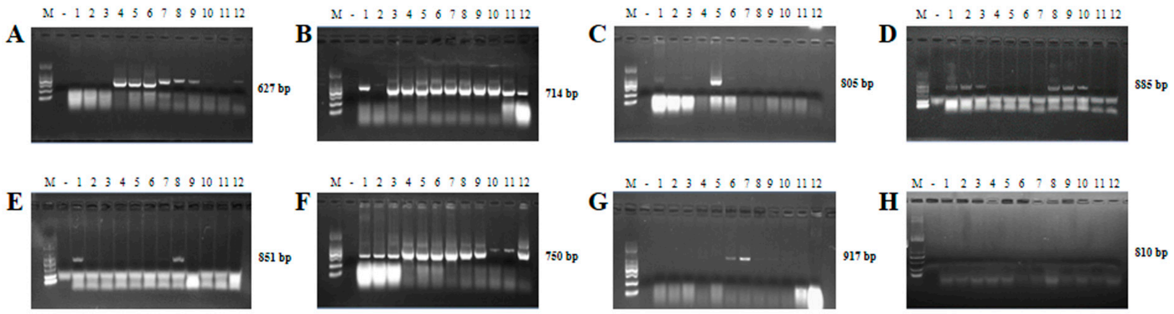

Figure S1.
